# Spontaneous Osteo-myelitis of the Long Bones

**Published:** 1880-01

**Authors:** N. Senn

**Affiliations:** Milwaukee, Chairman of Committee on Surgery


### Original Communications.

---

ARTICLE II.

SPONTANEOUS OSTEO-MYELITIS OF THE LONG BONES. BY N. SENN, M. D., of Milwaukee, Chairman of Committee on Surgery. Read before the Rock River Medical Society of Wisconsin, at its meetiing held in West Bend, Nov. 8, 1879, and published by request of the Society.

Since the appointment of your committee, no great innovations or improvements have been made in the department of medicine assigned to your committee, hence I ask your indulgence if in the selection of the subject for my report, I have disregarded the letter of our by-laws. I shall invite your attention to a consideration of "Spontaneous Osteo-myelitis of the Long Bones." While traumatic osteo-myelitis was carefully studied by our surgeons during the late war of the rebellion, the spontaneous variety has attracted but little attention, and is entirely ignored by most of our text-books. As this affection is quite prevalent in this section of country, and is at present but imperfectly understood by the majority of the profession, I trust a discussion of this topic on this occasion may prove to be interesting and profitable to us all.

*Definition.*—Spontaneous osteo-myelitis is an infectious inflammation of the marrow of bone. All inflammatory diseases of the bones involve, to a greater or less extent, the medullary tissue; practically the surgeon understands by this term an affection where the inflammatory process begins primarily in this substance by localization of the infectious poison.

*Synonyms.*—A great variety of terms have been applied to this disease at different times and by different authorities, as, endostitis, medullitis, ostitis, ostitis acutissima, osteo-myelitis diffusa, diffuse suppuration of bone, periostitis rheumatica, (diffusa, acutissima,) osteo-phlebitis, typhus des os, typhus des membres, pseudo-rheumatic inflammation of joints and bones. These different terms have served to convey the ideas entertained by different authors in regard to the location, nature or ætiology of this affection. Although every bone in the body containing marrow may become the seat of osteo-myelitis, the following remarks will apply to the disease only as it occurs in the long bones.

*History.*—Although this affection must have had an existence almost as early as disease itself, we find no mention of it until 1705, when J. L. Petit described an affection of the bones which was identical with what we now understand by osteo-myelitis. Similar allusions have been made to it by Gooch, Pott, Cheselden, Hey and Abernethy, some of their descriptions being sufficiently accurate to enable us to recognize the character of the lesion. The traumatic form was studied with more care and assiduity than the spontaneous variety. In 1831, M. Renaud published a paper "On Inflammation of the Medullary Tissue of the Long Bones," in which he gives a report of five cases occurring after amputation, all having terminated fatally. Cruveilhier alludes to this affection when he says, "The phlebitis of the bones is one of the most frequent causes of visceral abscesses following wounds or surgical operations in which the bones are involved. Roux credits Nélaton with having devised the term osteo-myelitis in 1834, and having published a brief account of it in 1844. In 1849, Mr. Stanley, in his excellent work "On Diseases of the Bones," gave an accurate description of the spontaneous variety under the title "Suppuration in Bone." In 1855, Chessaignac applied the term osteo-myelitis for the first time to the spontaneous form, reporting four cases. Traumatic osteo-myelitis has always been of frequent occurrence in military practice and in infected and badly ventilated hospitals. Among the military surgeons who have advanced our knowledge of this form of the affection, Vallette, M. Roux, Jules Roux, Larrey, Pirogoff, Lidell and Allen deserve prominent mention. In 1865, W. Roser gave a complete resumé in thirty propositions, of what was then known concerning the spontaneous variety. While the traumatic form was generally admitted to be of an infectious origin, the spontaneous type was considered purely inflammatory until Durcke ascertained its infectious character. Demme, Volkmann, Schede and Hueter have added valuable contributions to our knowledge of this disease by their pathological investigations and clinical observations. Rosenbach and Kocher, by numerous experiments on animals, have established many facts corroborating the theory advanced by Durcke. From the history of this disease, it will be seen that our knowledge pertaining to it is of comparatively recent date. Before pathological anatomy established the fact that the marrow is always the primary seat of inflammation, this affection was confounded with ostitis or periostitis. Before osteo-myelitis was recognized as a distinct affection, ostitis was regarded as a frequent disease of bone. At present osteo-myelitis must be considered the most frequent of all inflammatory diseases of bone, while ostitis is of very rare occurrence.

In opposition to the generally received opinion that the disease originates primarily in the medullary tissue, are the views of M. Gosselin and M. Guérin who attribute the suppuration in the marrow to an antecedent subperiosteal suppuration, occurring especially where the matter has not been evacuated by an early incision. They regard osteo-myelitis as a lesion secondary to inflammation of the substance of the bone proper, or its fibrous envelope, and seek to prevent it by free and early incisions, even to the extent of penetrating into the interior of the bone. M. Panas has found the myeloid tissue not only in the central canal of the long bones, but likewise in the deeper layer of the periosteum and the Haversian canals; it is therefore not necessary for the disease to originate in the central medullary canal, but it may, and very frequently does, commence at any point where myeloid cells are found. This view is substantiated by the clinical fact that this disease affects with greater frequency individuals during a time in their lives when the medullary tissues exist in greatest abundance, and by its manifesting a strong predilection for those portions of the bones containing the greater amount of marrow. From the peculiar relation of the structures it is easy to understand that inflammation of bone or periosteum will affect the medullary substance by contiguity, but the surgeon applies the term osteo-myelitis exclusively to those cases where the marrow is the primary focus of the inflammatory process.

*Structure and Function of Marrow.*—Periosteum, osseous tissues proper, and medullary tissue are anatomically distinct, but in a physiological sense combined, being supplied by the same nerves and blood vessels. Any one or more of these substances may disappear during pathological changes, and may, by metamorphosis of their histological elements, be converted into each other. A similar metamorphosis of those tissues constitutes a well established fact in physiology. By sympathy and contiguity a pathological or physiological process in one structure is very apt to involve others to a greater or less extent. The appearance and structure of marrow varies according to age and locality in which it may be found. In the fœtus and in the cancellated structure of bone it is red and contains only a trace of fat, while in the central medullary cavity of the long bones in the adult, it presents a yellowish color, and is composed almost entirely of fat. The difference in color depends on the presence or absence of fat. The red or embryonic marrow contains about 86 per cent. of myeloid cells. These are round simple embryonal elements measuring from 0.0129 to 0.0257 m. m. and containing one or two nuclei and granular matter. Some of these cells assume a spindle-shaped form and are transformed into connective tissue cells, others are changed into fat cells, while the rest preserve their lymphoid character. An important physiological function has been lately assigned to these bodies by Neumann and Bizzozero, viz.: the production of red and white blood globules which enter the circulation by immigration, a process opposite to the one described by Cohnheim. Neumann and Eales have observed a pecularity of the capillaries in the medullary tissue, their caliber being four times greater than that of the arterial branches that immediately supply them. This dilatation of the capillaries diminishes the rapidity of the blood current and favors the accumulation of the white globules of the blood and may possibly undergo here a transition from white to red corpuscles. The process of hæmato-genesis in the medulla may therefore be intra-vascular and extra-vascular, in the former case depending on a metamorphosis of the white corpuscles, in the latter instance on a transformation of myeloid cells into blood corpuscles. To explain the possibility of the red corpuscles entering the capillary vessels from without, Dr. H. C. Hand, of St. Paul, suggests that during the formation of lacunæ the vascular walls may suffer a slight loss of continuity, affording thus an ingress to these bodies. The blood-producing function of the myeloid tissue has also received attention in this country through the investigations of Dr. H. C. Hand, Prof. H. C. Wood and Prof. Wm. Pepper, and in the light of experiment, microscopical examination and clinical observation, must now be considered an established fact in physiology.

The yellow marrow found in the central canal of long bones is composed of 96 per cent. of fat. The fat vesicles are lodged in a nidus of delicate fibers of connective tissue, interlacing freely in every direction, and becoming more abundant towards the outer portions of the marrow. In a healthy bone there is no such thing as an endosteum; this had only an existence in the imagination of the older anatomists. Bundles of connective tissue fibers are arranged longitudinally with the bone, but no complete membrane is formed, and the medulla is in direct contact with the bone tissue.

The principal source of blood-supply is the nutrient artery, branches of which form a delicate network, with meshes of polygonal shape. Free anastomosis exists between the vessels in the central canal and the reticulated tissue.

The nerves are derived in greater part from the cerebro-spinal system, and enter the bone with the vessels, through the foramina nutritia and the Haversian canals.

The presence of lymphatics has been demonstrated by fine injections by Bichât, in the substance of bone, if not in the central medulla. During early fœtal life, marrow does not exist; it is formed by transformation of osseous into medullary tissue, so that during the development of bone, not only bone but medullary tissue is formed. The most important anatomical properties to be remembered in connection with our subject are: the softness, vascularity and exuberance of the cellular elements of the medulla, as well as the unyielding character of the walls surrounding it, all important factors in the production of inflammation and infection.

*Ætiology.*—Infection is the essential cause of traumatic, as well as idiopathic osteo-myelitis, the difference consisting in the manner in which infection is produced. In the traumatic form the infecting element is brought in direct contact with the marrow through a wound after compound fracture, amputation or resection; in the spontaneous form the poison enters the circulation, and is carried with the blood to the medulla, where it becomes localized.

The influence in preventing traumatic osteo-myelitis in contaminated hospitals has been strikingly illustrated in the general hospital in Munich. Before Lister's treatment of wounds was adopted, almost every patient who was brought to the hospital with a compound fracture, or had submitted to an amputation or resection, became affected with this disease, and the majority of them died of pyæmia. Since the introduction of the antiseptic treatment, this dreadful scourge has been unknown in that institution. The spontaneous variety is met with in a large proportion of cases in individuals under 25 years of age, before ossification of the epiphyseal cartilage has taken place. The period of growth and development of bone constitutes the most important predisposing cause. Perhaps 90 per cent. of the whole number attacked are between five and fifteen years of age. I have seen at least three cases where the patients were more than thirty years of age.

CASE I. *Osteo-myelitis of femur, with secondary suppurative synovitis of knee-joint; pyæmia; death.*—Joseph S.. aged 38, a laborer by occupation, in previous good health, received a slight injury of the lower part of his right thigh by a fall during the month of September, 1872. The injury was soon forgotten, but in a few days the patient experienced severe pain in the lower part of the thigh, attended by febrile disturbances, followed by a diffuse swelling. Three weeks from the beginning of the attack, the knee-joint became swollen, and the general symptoms assumed a more serious aspect. I saw him about five weeks from the time the first symptoms had developed themselves, and found him very much emaciated and suffering from hectic fever. Several openings existed over and above the knee-joint, discharging profusely their sanious pus ; a probe could be passed readily into the joint and into the interior of the femur, coming everywhere in contact with denuded bone. Amputation of the thigh was advised as the only means to save the patient's life, but was not acceded to, and the patient died two weeks subsequently, with symptoms of pyæmia. Cough, rapid breathing, chills and a jaundiced hue of the conjunctiva and skin made their appearance a few days before the fatal termination.

In this case the morbid process commenced undoubtedly in the lower and interior part of the femur. After suppuration had taken place, pus infiltrated the spongy portion of the condyles, and invaded the knee-joint through a perforation in the articular cartilage of the joint, exciting there a suppurative synovitis.

An early incision, with perforation of the compact layer, affording an outlet for the pus, might have been the means of saving the patient's life and limb.

CASE II. *Osteo-myelitis of lower extremity of tibia ; necrosis; operation ; recovery.*—August Musach, aged 35, laborer, with a scrofulous hereditary taint, came under observation in the fall of 1872. When he was about 12 years of age he received an injury to the lower portion of the right tibia, acute inflammation followed, resulting in suppuration and spontaneous evacuation above the internal malleolus. The opening discharged pus for a long time, when it finally closed, leaving the bone enlarged but causing no further inconvience. About a year ago he injured the same place with the blunt edge of an ax. Soon after the accident the part became very painful and in a few days swollen ; in about five weeks a physician opened an abscess over the minor and lower aspect of the tibia. The pain and fever subsided but a thin and extremely offensive pus continued to escape until I saw him. At that time several openings existed above the internal malleolus; the lower end of the tibia was enlarged to more than twice its normal size. A probe passed readily through one of the cloacæ and came in contact with loose, dead bone. The soft tissues were very much indurated. The patient appeared to be well nourished but very anæmic. He was etherized and the bone exposed by an incision. The cloaca was enlarged with the trephine and chisel and a sequestrum of cancellated bone infiltrated with fœtid pus was removed. The involucrum was fully an inch in thickness and almost as dense as ivory. The abscess cavity was lined throughout with large, whitish-grey granulations; only a very thin shell of bone seemed to separate the abscess from the anklejoint. The patient made a slow but permanent recovery. In this case the disease during childhood affected the lower epiphyseal cartilage and the spongy tissue on both sides of it and its results undoubtedly furnished a predisposing element for the second attack.

CASE III. *Suppurative synovitis of knee-joint; osteo-myelitis of femur; death.*—John Schulz, aged 32, laborer, with a hereditary tubercular taint suffered during his childhood from a synovitis of the left knee-joint, which terminated in false anchylosis and partial dislocation of tibia without suppuration. While at work during the spring of 1878 he injured the affected knee by a fall. In a few days severe pain and swelling supervened, accompanied by fever. A number of weeks afterwards an abscess appeared on the inner side of the knee, which was opened by a physician. When I saw him, about eight months from the commencement of the disease, I found the patient very much emaciated, an opening on the inner side of the knee discharged freely a sanious pus and permitted the passage of a probe into the joint when denuded bone was distinctly felt. The joint was swollen, tender and extremely painful on the slightest attempt to move it. I proposed to make a free incision on both sides of the joint, and, with a sharp spoon scrape away the carious articular ends under antiseptic precautions. I failed, however, to obtain the consent of the patient and his friends, and lost track of the case until about four months afterward, when I was again called to see him. I then learned that the whole thigh became swollen and extremely painful about three weeks previous to my visit. Several openings existed over the inner and outer aspect of the thigh, through which a probe could be passed down to the femur, which was found denuded of periosteum. The patient was suffering from chills and presented a jaundiced appearance. The spleen was considerably enlarged and over the posterior and inferior part of the chest numerous râles could be heard. As internal metastatic deposits had evidently taken place, only a palliative treatment was adopted. The patient succumbed rapidly to the general invasion and in a few days death terminated his sufferings. In this case osteo-myelitis occurred as a secondary affection following destructive suppurative synovitis. The disease in the joint had destroyed the articular cartilage, and, from imperfect drainage, the pus in the joint infiltrated the reticulated structure of the condyles of the femur, causing finally a diffuse and fatal osteo-myelitis. It is evident that the establishment of free drainage, combined perhaps with *évidement* under antiseptic precautions, might have prevented the extension of the disease to the femur.

Location and climate appear to exert an influence in the causation of this disease. According to Volkmann it is frequently met with in Berne, Marburg, and Halle, but seldom in Zürich and Berlin. From the great number of patients suffering from necrosis and from the number of recent cases that have come under my observation, I am inclined to believe that this disease is quite prevalent in our section of country, more particularly along the lake shore. The frequent and sudden changes in the temperature, incident to this region, are a fertile cause in the production of internal congestions.

"Mr. Savory reports a case in which the exposure of a limb to sudden extremes of heat and cold by plunging it into ice cold or very warm water, caused acute osteo-myelitis and McNamara, of Westminster Hospital, cites the case of a boy in perfect health who fell through the ice Dec. 20th and on Feb. 15th suffered amputation at the knee-joint in order to prevent further absorption by extension of evident septicæmia. Dissection of the limb showed that the cancellated tissue and medullary canal of the tibia were entirely destroyed and that the outer shell (compact tissue) of the bone was more healthy than any other part; the myelitis being evidently not due to extension of inflammation from the periosteum to the medulla through the Haversian canals, as might naturally be supposed."

I have seen a number of cases where the exciting cause could be clearly traced to a sudden chilling of the cutaneous surface.

CASE IV. *Osteo-myelitis of humerus; necrosis; operation; recovery.*—Henry R., aged 18, farmer, previously in perfect health, was attacked early in the spring of 1865, with severe pain in the upper portion of his humerus, almost immediately after bathing in cold water. He had over-heated himself by a long and rapid walk before he went into the water. The fever and general disturbance were severe from the beginning. After three days a diffuse swelling took place, extending over the whole arm and part of the forearm. In about three weeks, after the most terrible suffering, an abscess formed and discharged itself near the insertion of the deltoid muscle. Several other openings formed subsequently, through which several small pieces of bone escaped. The movements of the shoulder-joint became impaired and the muscles in its neighborhood became atrophied. At the end of the operation, June, 1871, several cloacæ existed, through which a loose sequestrum could be distinctly felt with a probe. The involucrum was found exceedingly dense and measured over an inch in thickness. The sequestrum was rough on all sides and had evidently a central origin. The primary disease involved the interior of the bone, and had extended from or over the upper epiphyseal cartilage to the middle of the humerus. Judging from the impaired motion in the shoulder-joint, the previous synovitis must have been quite severe. In about four months the wound had entirely healed, and the usefulness of the arm had materially improved.

CASE V. *Osteo-myelitis of tibia and radius; recovery.*—Reinhold Stolz, aged 12 years, a healthy boy, after over-heating himself in a game of ball, rested himself by lying on the cold ground until he became thoroughly chilled. When he came home he complained of a severe pain in his left leg, and the next day he was unable to walk. On my visit at this time (Oct. 30, 1877), his pulse was 140, temperature, 40°. The lower part of the tibia was tender, but no swelling could be detected. The least attempt to move the limb caused intense pain. In a few days a diffuse phlegmonous swelling appeared and an incision gave exit to a considerable quantity of reddish brown fluid, in which free oil globules were found floating on the surface. The tibia was found denuded of periosteum over several inches of its extent. The pain continued after the incision, and the general symptoms assumed a more serious aspect. During the second week of the disease the lower part of the radius on the right side became swollen and tender; suppuration did not take place; the pain and swelling yielded in a few weeks to the persistent local use of tincture of iodine.

After a few months the general symptoms improved, and when last seen, the patient was able to walk, but the lower end of the tibia was found to be the seat of extensive central necrosis.

Over-heating of the body diminishes its power to resist the deleterious influences of cold. The internal congestion resulting from a sudden abstraction of heat from the cutaneous surface produces a stasis in the capillaries of the medullary tissue which favors the localization of the infectious germs which may exist in the blood. The influence of temperature is further apparent from the fact that, according to Luecke and Schede, the disease seldom occurs during the summer months. My observations would tend to corroborate this assertion. Most of my patients contracted the disease during the months from October to May.

Among the general predisposing causes may be enumerated, the period of youth, the male sex, occupation, a damp and changeable climate, scrofula, syphilis, and the period of convalescence after acute infectious diseases. Crowded school-rooms, badly ventilated sleeping apartments, damp dwelling houses, and other unfavorable hygienic surroundings, are potent agencies to render the system susceptible to infection. Among the local causes may be mentioned fractures, contusions and concussions, but traumatism in every form can only be viewed in the light of exciting causes. No amount of local mechanical, physical or chemical irritation is sufficient to produce the disease independent of the essential cause—infection. Rosenbach has even gone so far as to assert that the infection is one *sui generis*, a specific infection, while Kocher and most of the modern pathologists regard the infection analogous in charater to that producing other forms of inflammation. Both of these observers have made numerous and ingenious experiments on animals to prove the infectious nature of this disease. Rosenbach found that contusions, fractures and crushing of bone, as well as destruction of marrow by cauterization, artificially produced under antiseptic precautions, were insufficient to produce osteo-myelitis.

In eleven cases of cauterization of the central medulla with a galvano-caustic wire, under the antiseptic treatment; in eight no suppuration took place; in three, where the burning was severe, necrosis occurred; the general symptoms were not severe, and suppuration very slight. In three cases where the marrow was operated upon by electrolysis, the results were negative. Injections of mercury, amalgamate of sodium, caustic potassa and sulphuric acid, directly into the medullary cavity, caused instant death during the operation, from the entrance of the irritants into the veins, but osteo-myelitis failed to be produced in any of them. Injection of a mixture of equal parts of croton oil and fat, previously heated to 100° C., produced intense inflammation of the soft parts, destruction of medulla and complete necrosis of the shaft of the bone. Two drops of pyæmic fluid, injected into the interior of the tibia of a rabbit, caused death in three days. An examination of the bone showed that extensive osteo-myelitis and necrosis had taken place. Rancid butter injected into the medulla excited a severe phlegmonous osteo-myelitis which came near proving fatal. On killing the animal some time afterwards, the entire diaphysis was found necrosed and separated from both epiphyses. In three cases where pus from an osteo-myelitic patient was injected into the medulla, well-marked, typical osteo-myelitis followed. These experiments were made on dogs and rabbits.

From the results of these experiments, Rosenbach concludes that mechanical, physical or chemical irritants applied to the marrow, with the exclusion of infection, cause simply death of its substance and the subsequent removal or absorption of the dead tissues. Injections of infectious matter excite a diffuse, purulent, phlegmonous, septic inflammation, followed in some instances by general infection and death. Osteo-myelitis, traumatic and spontaneous, can only occur after infection has taken place, no matter how severe and extensive the local destruction may have been. In spontaneous cases the infectious material circulates in the blood, and localizes itself in the medulla, where it reproduces itself, causing septic inflammation. Localization of the poison in many instances is determined by local causes in individuals laboring under general infection.

Luecke assumes that a stagnation in the capillaries of the marrow, from any cause, may arrest the infectious germs at the point of obstruction where they find a fertile soil for their reproduction. Intense infection may lead to a fatal termination without localization. In most cases the poison remains latent or innocuous in the system until some local cause or causes determine its localization.

Kocher, who does not believe in the specific nature of the infection, was unable to produce, artificially, osteo-myelitis in animals by injecting into the marrow pus obtained from patients suffering from this disease. He regards osteo-myelitis, not as a specific infectious disease in the same sense as typhus, scarlatina and diphtheria, but simply infectious in the same sense as every other acute inflammation.

His experiments on dogs, by applying directly to the marrow chemical or mechanical irritants, resulted in ostitis circumscripta hyperplastica; osteo-myelitis did not occur in a single instance, provided infection was prevented. Injections of septic fluids containing bacteria produced the disease promptly. Injections of pure pus, with micrococci, was followed by sclerosis of the bone. Injections of caustic ammonia, and feeding the animal afterwards with decomposed blood, mixed with Pasteur's solution, resulted in osteo-myelitis, with grave constitutional symptoms. He believes also that traumatic influences are insufficient to produce osteo-myelitis without the aid of local or general infection. In external wounds communicating with the marrow, the septic germs in the air are brought in direct contact with the marrow; where no such direct entrance is possible, the septic bacteria enter the circulation through the gastro-intestinal or broncho-pulmonary mucous membrane, and with the blood-current reach the medulla.

Schueller succeeded in producing osteo-myelitis in animals by subcutaneous injections of decomposed blood. Mikulicz has ascertained that the presence of living micro-organisms in the septic fluid is essential for the production of progressive sepsis after injection; only slight reaction took place if the germs were destroyed by the addition of glycerine. S. Hasse found that a much smaller quantity of osteo-myelitic pus would produce a more intense effect on the same dog if it was allowed to undergo decomposition for six days. When the circulation of the blood in the marrow is impeded, or extravasation has taken place, the septic organisms circulating in the blood are readily arrested, and find there a fertile soil for their development and reproduction.

Klebs, Recklinghausen and Langhaus have found micro-organisms in osteo-myelitic patients, in the inflamed medulla, in the secondary deposits, the thrombi and fat emboli, in the fibrous exudations on serous membranes, and in the lymphatic vessels. In not rare instances, according to Billroth, diplo-cocci and strepto-cocci were also found. In some cases the micrococci were so numerous as to constitute the principal bulk of the products of inflammation, imparting to the fluid a reddish-brown, emulsion-like appearance. The following cases may be of interest in this connection:

CASE VI. *Osteo-myelitis of femur; suppurative synovitis of knee-joint; micrococci found in pus.*—Albert P., aged eight years, had been in his usual health until September 10, 1879. While at school, he was attacked almost suddenly with a severe pain in the lower part of the thigh, which was attended by several chills and fever. On the second day the pulse was 140, temperature 40°; tongue coated; total loss of appetite; great thirst. The pain was very severe. Great tenderness, but no swelling over the lower epiphyseal cartilage of right femur. He was very restless and soon became delirious. Salicylate of soda in large doses was administered and tr. iod. and ice applied over the lower part of thigh. On the fourth day the knee and lower part of thigh commenced to swell. On the tenth day deep fluctuation could be felt; the swelling appeared to terminate somewhat abruptly about the middle of the thigh, the surface pitted on pressure, and the superficial veins were enlarged. Chloroform was administered and a free incision was made on the outer aspect of the thigh between the quadriceps extensor femoris and biceps muscles; after dividing the skin and fascia, the handle of the scalpel was used to separate the muscles until the periosteum was reached. This membrane presented a dark brown almost black color; fluctuation was distinct underneath it; it was incised freely, and about two ounces of a thick brownish red matter escaped. On introducing the finger the femur was found completely denuded of its periosteum from the lower epiphyseal line upwards to the extent of about six inches. The epiphyseal line was marked by a deep groove indicating beginning epiphyseolysis. The operation was performed under antiseptic precautions.

A microscopical examination of the matter removed, made immediately after the incision, showed the presence of red and white blood corpuscles, pus globules, fat globules and numerous living cocci. After the incision the pain and fever became less; both re-appeared with increased violence after swelling of knee took place. A large aspirator needle was introduced into the joint, and a small quantity of pus removed; on injecting a five per cent. solution of carbolic acid, it was found that the fluid escaped through the incision above, demonstrating the fact that the knee-joint was in direct communication with the primary seat of osteomyelitis in the femur. Marked improvement followed this procedure. A splint was applied to support the parts and prevent if possible, a complete separation of the lower epiphysis. In this case the morbid process began near the cartilage of agglutination and became arrested at the middle of the femur above; below, the pus escaped at the partially separated epiphyseal junction, but subsequently infiltrated the epiphysis and reached the joint through a perforation of the articular cartilage. The most favorable result that can be hoped for in this case, will be extensive necrosis of the lower portion of the shaft of femur and partial or complete anchylosis of the knee-joint.

The femur is most frequently affected. Seventy-three per cent. of all of Demme's cases involved the femur. In this bone the disease manifests a special preference for the lower epiphysary region, while in the tibia the order of frequency, is reversed. The great frequency with which the extremities of the shaft of the long bones are affected, receives a plausible explanation from the activity of the physiological changes during the growth of bone, and perhaps to a lesser extent by the greater frequency of traumatism in these localities.

English claimed that the extremity of the shaft and epiphysis toward which the nutrient artery is directed, is always primarily affected, on account of the greater blood pressure in that locality. Clinical experience has proved the contrary.

Volkmann has called attention to embolism as a cause of osteo-myelitis and necrosis.

Ritter reports a case of spontaneous fracture of clavicle following osteo-myelitis, which was caused by embolism of the principal vessel supplying the bone. The patient was a woman 34 years of age, who, four days after an abortion, suffered from a periosteal abscess over the middle of the clavicle, which was opened by incision and closed in four weeks. A painful spot remained over the seat of the abscess. At this point a spontaneous fracture took place after which the pain disappeared. Union with deformity took place in the usual length of time.

Azam reports a case where osteo-myelitis followed tumor albus of the knee in a patient 32 years of age.

Case III affords a similar illustration.

Billroth has seen several cases of osteo-myelitis follow acute articular rheumatism of the knee-joint.

Stanley has observed several cases of suppuration of bone near the articular ends with implication of the adjacent joint, caused by tubercular deposits in the cancellæ, as tubercular matter was found in the purulent discharges.

*Symptomatology.*—The symptoms attending osteo-myelitis always indicate a grave affection. They vary in intensity according to the location and extent of the disease and later in the disease they are modified by the complications that may arise. In severe cases a chill or succession of chills followed by high temperature initiates the disease. The febrile state sometimes obscures the local symptoms to such an extent as to lead the physician to suppose that the patient is suffering from an attack of typhoid fever. If we recollect that Lenator entertains the opinion that osteo-myelitis may occur during an attack of typhus fever, the difficulty in diagnosis must be very great in some instances as the latter would obscure the local manifestations. Goltdammer reported a case of acute osteo-myelitis of the tibia to the Medical Society of Berlin, where the general symptoms simulated typhoid fever to such an extent that the patient, after an illness of ten days, was sent to the medical wards as a severe case of typhus fever.

The patient had a pulse of 110–120, temp., 40.41° C., tympanitis, furred tongue, enlargement of the spleen, bronchitis, rapid respiration and delirium. On close examination, a slight swelling was found over the lower part of the right tibia with tenderness on pressure which led to the diagnosis of acute osteomyelitis. During the progress of the case, pleuritis, parotitis duplex and synovitis of the right shoulder-joint made their appearance. The patient died 8 days after admission, or 18 days from the beginning of the disease. A post mortem examination revealed the existence of acute osteo-myelitis of the tibia and pyæmia. Chassaignac believes that diarrhœa is present in almost all cases in the beginning, but it is a more constant symptom after septicæmia or pyæmia have made their appearance. Usually the first symptom is the pain. This is generally severe, deep-seated, constant, boring, tearing or throbbing and referred to the primary center of the disease, usually in the vicinity of an epiphysary cartilage. Patients old enough to describe their sensation, complain of a feeling as if the bone was being broken. The patient objects to moving or handling the limb on account of an aggravation of this distressing sensation. In some instances where the disease begins insidiously the patient may continue to walk for several days, but is lame on account of the pain, which is increased by the weight of the body making pressure upon the cartilage of agglutination. This aggravation of pain, by bringing the epiphysis and diaphysis in forcible contact, is an early, prominent and important symptom. Impairment or entire loss of function of the nearest joint and the entire limb is one of the first symptoms. Swelling seldom takes place before the third day, but tenderness is present from the beginning and is usually located at the junction of the diaphysis and the epiphysis, and, from the close proximity of this point to the joint, disease of the latter is generally suspected by the patient and not unfrequently also by the physicians. In chronic cases, swelling may not take place for weeks or months ; when it does, it is due to an extension of the morbid process to the periosteum or the neighboring soft parts. After it has once made its appearance it increases with great rapidity, including usually one joint and extending over a surface corresponding to the extent of bone involved. The swelling is œdematous and terminates abruptly at a point where the disease in the interior of the bone has become limited. Chassaignac was the first one to call attention to this peculiar clinical fact and place great stress upon its diagnostic significance. The superficial veins as a rule are enlarged and if the obstruction in the deep-seated veins from thrombosis or pressure becomes great, œdema of the entire limb may take place. In regard to fluctuations, Ed. V. Wahl says that it is circumscribed at first in phlegmonous inflammations of the soft parts, while it is diffuse from the beginning in osteo-myelitis. I regard this distinction as a good one.

From the anatomical location of the primary seat of the lesion it is easy to conceive that the products of inflammation are evacuated spontaneously only after extensive destruction of bone and soft tissues. On account of the resistance offered by the unyielding walls of the compact tissues many of the products of inflammation are forced into the circulation through the veins, producing in the first instance high temperature, and, later, fat embolism and pyæmia. Numerous experiments made with injections of chemical and medicinal substances directly into the marrow, have proven the possibility of thus forcing the substances directly into the veins. It is therefore logical to assume that affections of veins in cases of osteo-myelitis must be of frequent occurrence. In most of the fatal cases where post mortem examinations have been made, the veins were found affected, their interior being filled with thrombi in different pathological conditions. These necessarily lead to metastatic deposits in various internal organs. Fat embolism and pyæmia take place in a similar manner; both are attended with difficulty of respiration, cough and an aggravation of the general symptoms. In many severe cases the morbid process is not limited to a part or an entire bone, but appears simultaneously or in rapid succession in different bones of the body. Cases of this description have induced Roser to apply the term pseudo-rheumatism, and Simon used the term necrosial fever from their resemblance to acute articular rheumatism. Volkmann regards this form as a rare affection and applies to it the term osteo-myelitis spontanea multiplex. A number of cases of this, the severest form of osteo-myelitis, have come under my observation.

CASE VII. Helen M., aged 18, not affected with any constitutional taint, was in good health until Nov. 12, 1872, when after a supposed exposure to cold she was taken suddenly ill with a chill, followed by high temperature (40.5°), and pain in the right knee and left shoulder-joint. The pulse varied between 120–140, and the fever was continuous; the morning and evening temperature ranging between 40°–40.5°. The general symptoms, and especially the low muttering delirium simulated typhoid fever, and almost obscured the local symptoms. Diarrhœa, slight cough, and rapid breathing were early and conspicuous symptoms. The pain in the localities mentioned, was at first severe but soon subsided after the appearance of a diffuse œdematous swelling. Well-marked tenderness existed over the upper epiphysary region of the tibia and humerus. New points of inflammation made their appearance during the progress of the case, at the middle of the clavicle on right side; lower end of femur, left side; third and fourth rib, left side; lower end of radius on right side. At the end of the first week deep fluctuations could be detected at most of these places, but the patient's general condition had assumed such a serious aspect that no incisions were made. Notwithstanding the early and persistent use of stimulants and tonics, the patient died on the 10th day of the disease, with symptoms indicating serious lung complications from metastatic deposits.

CASE VIII. *Multiple osteo-myelitis; pyœmia; death.*—Joseph E., aged 14 years, a healthy boy, was taken Dec. 31, 1878, with a severe pain in the middle of the right thigh, which rendered him at once unable to walk. The thigh did not become swollen for three days. Chilliness and fever were present from the beginning. When first seen the thigh was swollen and œdematous from the lower third upward, measuring two inches more than the opposite one; superficial veins enlarged; pulse 120; temperature 40°. The patient was very restless and delirious most of the time; great thirst and total loss of appetite. On the ninth day the lower part of the right fore-arm, on radial side and wrist was found swollen, as well as the right parotid region, the latter occurrence rendering deglutition difficult. He remained in about the same condition, the pulse ranging from 116 to 130, and the temperature from 38.5° to 40° until the 21st when the left knee commenced to swell; December 30th, a swelling appeared over the left clavicle; January 15, 1879, fluctuation being present, an incision was made on the outer aspect of the thigh, lower part of radius and middle of clavicle, giving exit to a thin sanious fluid. The femur and radius were found extensively denuded and the clavicle had suffered spontaneous fracture about its middle. The abscess cavities were kept open by drainage tubes and were washed out daily with a 3 per cent. solution of carbolic acid. Toward the final termination the patient became sallow and suffered from a dry hacking cough, symptoms plainly indicating the presence of pyæmia. The patient died on the 27th of January, eight weeks from the beginning of the disease.

CASE IX. *Multiple osteo-myelitis; necrosis; recovery.*—John S., aged 14, with an hereditary strumous diathesis, was attacked suddenly during the month of November, 1872, with a severe pain in the upper part of the right thigh and the lower part of the left tibia almost simultaneously, attended by violent constitutional disturbance, fever and delirium. In a few days a rapid and diffuse swelling appeared in both localities, in the thigh it evidently involved the hip-joint. About two weeks afterward, the patient failing rapidly, chloroform was administered and an incision made on the right side, extending into the hip-joint, which gave exit to a large quantity of a thick brownish red fluid, mixed with laudable pus. The upper portion of the femur was denuded of periosteum and the hip joint affected with suppurative synovitis. The incision over the lower portion of the left tibia liberated a moderate amount of a thin sanious fluid, mixed with numerous fat globules. From the epiphysary junction upward, the tibia was separated from the periosteum for a space of more than five inches. The abscess cavities were washed out daily with carbolized water, and nutritious food was freely administered as well as tonics and stimulants. The pain diminished after the incisions, but the general condition did not improve for several months. Profuse suppuration continued for several months until the upper epiphysis of the femur and a number of spiculæ of bone escaped spontaneously from the hip-joint as well as a sequestrum from the tibia when it almost ceased and the patient regained his former health. In about one year the wounds had closed completely. The left leg was perfect; the right showed nearly two inches of shortening, but a movable false joint with slight flexion of the thigh upon the pelvis.

In some cases the disease appears in another, perhaps distant bone in a sub-acute form several weeks after the first invasion, and fails to develop itself fully because the original infection appears, as it were, to have expended its force at the primary seat of localization. In such cases the process terminates with an increased production of bone instead of suppuration. To substantiate this assertion I will report the following cases:

CASE X. *Osteo-myelitis of femur; spontaneous fracture; hyperplastic osteo-myelitis of radius; recovery.*—August Schulz, aged 13, son of healthy parents, after an alleged fall, was taken with severe pain in the left thigh during the winter of 1874, which soon rendered walking impossible. I saw him two weeks afterwards, and found the whole thigh and knee enormously swollen and œdematous. The superficial veins were enlarged. Fluctuation was perceptible around the whole circumference of the thigh, and was well-marked on the outer aspect of the limb, at the junction of the lower with the middle third of the femur. The patient was emaciated and very anæmic. An incision was made between the biceps and quadriceps extensor femoris, about 5 ctm. above the knee-joint, which gave exit to a large quantity of a mixture of a thick, brownish-red emulsion, with pus. The lower end of the femur, from the epiphysary junction upwards, to the extent of 20 ctm., was found completely denuded of periosteum. Drainage tubes were inserted, and the cavity well washed out daily with carbolized water. About two weeks later I found, during one of my visits, that the femur had fractured spontaneously at a point near its middle. This occurrence added greatly to the perplexities in the management of the case, and increased my apprehensions in regard to the prospects of the future usefulness of the limb, and not less concerning the possibilities of saving the life of the patient. The limb was placed as nearly as possible in its natural position, with the knee slightly flexed, and a fenestrated plaster of Paris splint applied from the toes to the hip-joint. Soon after this unfortunate complication had taken place, the patient complained of a pain in the lower part of the right radius, and in a few days a circumscribed hard swelling made its appearance in that locality. Tincture of iodine was freely applied, which soon arrested the further progress of the disease and subdued the pain. About three months after the application of the first plaster splint, the new bone had become sufficiently firm to retain the position of the limb. The swelling above the wrist gradually disappeared, and the patient began to improve in his general health, so that during the following summer he was able to go to school with the aid of one crutch, a fistulous opening at the point of incision discharging a moderate quantity of pus. Three years afterwards I removed by sequestrotomy a large, rough, central sequestrum 17.5 ctm. in length, after which a permanent recovery took place, with a good, moveable knee-joint. Notwithstanding a slight curvature of the femur, with the concavity inwards, it is about 1.8 ctm. longer than the opposite one.

Case V may also serve as an illustration in this connection.

*Diagnosis.*—Mr. Holmes has well said that the disease under consideration is more frequently recognized at post mortem examinations than at the bedside of the sick. I am well satisfied that it is often mistaken and treated for other affections, as periostitis, ostitis, inflammation of joints, rheumatism, typhoid fever and even phlegmonous inflammation of the soft parts. When we remember that periostitis, ostitis, synovitis and cellulitis are secondary lesions intimately associated in the clinical history of every case of osteo-myelitis, and furthermore, that the fever attending it assumes such close resemblance to typhoid fever, it is not surprising at all that many cases are not correctly interpreted during life. A careful consideration of every feature of the clinical picture presented by each case, can only enable us to arrive at correct conclusions. There is no single pathognomonic symptom that would infallibly lead us to a correct diagnosis. The presence of fat globules in the products of inflammation was regarded as diagnostic by Chassaignac and Roser. Although this symptom is present in some of the cases, it is absent in a larger number, and it may also be present during the course of other suppurative processes, as I have repeatedly observed. Fat is almost always present in the purulent discharges from gangrenous inflammation of the cellular tissue. In a diagnostic point of view, the pus derived from the primary seat of the disease presents the following elements for examination: 1. Blood corpuscles, red and white, their number depending upon the acuteness of the attack. 2. Pus globules. 3. A thick, brown, emulsion-like fluid containing fat globules and micro-cocci.

One of the most important symptoms for differential diagnosis is the absence of swelling for the first few days regardless of the severity of other symptoms, also its rapid diffusion after it has once made its appearance. In periostitis and phlegmonous inflammation of the cellular tissue, swelling is one of the earliest symptoms. In osteo-myelitis the swelling is at first œdematous, extends symmetrically around the entire bone and terminates abruptly at a point where the morbid process in the interior of the bone has become arrested. Fluctuation in acute cases appears about the end of the first or during the second week. The superficial veins are usually enlarged. A consecutive affection of one or more joints in close proximity usually makes its appearance about from the end of the first to the fourth week. The time of its appearance as well as its character depends largely upon its causation. While joint affections are almost constant in osteo-myelitis, they are seldom associated with periostitis. Early incisions are of great diagnostic value; besides affording an opportunity of examining the condition of the bone, the subsequent history is of importance in arriving at a correct conclusion. If the pain and fever subside after the incision, we have to deal, in all probability, with a case of cellulitis or periostitis; if they are only diminished or continue unabated, it may be safely concluded that the disease is located in the interior of the bone. The frequency with which pyæmia occurs during the progress of this disease, must also be remembered in rendering a final diagnosis. A very interesting case was reported by M. Auger at a meeting of the Société de Chirurgie, on January 8, 1879, which well illustrates the obscurity of diagnosis in some cases, as well as the liability of neighboring joints to become involved if the disease occurs in an adult. The patient, a man 54 years of age, after having been out hunting, felt on the following day a violent pain in the right leg. The pain was most severe in the calf of the leg, the ankle and along the course of anterior tibial nerve, and came on in paroxysms without any regular intermittence. For a month the tibia was not swollen, when in a few days a purulent discharge took place on the upper third of the bone. Soon after this time the knee began to swell and the medullary canal was found filled with pus. An incision was made into the knee-joint on both sides under antiseptic precautions, and free drainage established. In three months the patient died of pyæmia.

*Prognosis.*—The mortality attending osteo-myelitis appears to be greater in hospital than private practice. Demme lost 4 out of 17 cases; Luecke, 11 out of 24; Kocher, 9 out of 26; and Schede 3 out of 23 cases.

Uncomplicated cases do not prove fatal very often. Roser says a fatal issue may take place without localization, and attributes death in such cases to an acute dyscrasia. Acute fatal cases can usually be traced to pyæmia, the result of phlebothrombosis. The organs most frequently affected by metastatic deposits, are the lungs, and next in frequency, the kidneys; in the former causing pleuritis, pneumonia or abscesses; in the latter, diffuse nephritis and anasarca. Fat embolism of the lungs has already been referred to as a cause of death. Great enlargement of the spleen is one of the gravest prognostic symptoms. Where the marrow is severely and extensively affected leucæmia is sometimes produced as a consequence, and of course leads to a fatal termination.

Schede has found the proportion of the white to red corpuscles of the blood change from 1.100 to 1.200.

I have invariably observed early and marked anæmia in all of my cases.

The thermometer furnishes us with an important prognostic aid in this as well as in many other acute affections.

If the morning and evening temperature remain constantly high, that is to say, range between 40°–40.5° during the first week, it indicates a severe case.

The more the general symptoms resemble typhoid, the graver the case. Chronic pyæmia is sometimes the cause of death. After incision secondary infection may take place, which may result in death by septic or pyo-septic infection. This result is more likely to follow in this than most other affections, as the cavities in the bone are reached and disinfected with the greatest difficulty.

In regard to the function of the limb after osteo-myelitis, a few words are necessary. Necrosis to a greater or less extent is the rule. Joint affections and partial or complete separation of epiphysis are frequent complications. If the effusion into the joint is passive from phlebothrombosis, or catarrhal, it is generally absorbed, and the function of the joint is restored completely. If the effusion is sero-purulent and the cartillage remains intact, aspiration and washing out the joint with a five per cent. solution of carbolic acid may be sufficient to restore the usefulness of the limb. Stiffness of the joint to a greater or less extent as well as mal-position of the articular ends of the bones, are events that cannot be avoided in all cases, even by the most skillful and attentive treatment. If the cartilage of the joint has become destroyed by suppurative synovitis, false or complete anchylosis and more or less deformity cannot be prevented. Separation of the epiphysis may be repaired completely, as will be seen from the following cases :

CASE XI. *Osteo-myelitis of both tibiæ; sero-purulent effusion in knee-joints; separation of epiphyses ; recovery.*—Carl Schulz, aged 14, after exposure to cold was taken suddenly with severe pain in the upper part of both legs during the winter of 1874. No swelling could be discovered the first four days ; after this time the upper part of both legs became rapidly swollen and œdematous. The patient was treated for rheumatism for four weeks. When I saw him, about this time, I found him very much emaciated and extremely pale ; pulse very rapid ; profuse perspiration, etc. A fistulous opening existed on both sides about three inches below the knee-joint, discharging freely this sanious pus. The legs were œdematous and the superficial veins enlarged. The shaft of the tibia was completely separated from the epiphysis on both sides. A probe could be passed through the opening between the diaphysis and epiphysis. The shaft of the tibia was denuded of periosteum over a considerable extent. Both of the legs were flexed and the slightest attempt to move them was attended by great pain. Both knee-joints were swollen. The patient was placed under the influence of chloroform and the existence of epiphyseolisis verified by the existence of preternatural mobility and crepitus at the epiphyseal junction. To ascertain the character of the effusion into the joints, aspiration was performed and a sero-purulent fluid removed. Both joints were thoroughly washed out with a five per cent. solution of carbolic acid and a plaster of Paris dressing applied from the toes to the middle of the thighs, with fenestrated openings over the anterior aspect of the leg. The abscess cavities were washed out daily with carbolized water and the wounds covered with cotton saturated in carbolized oil. Tonics and stimulants were given freely to support the patient's strength.

With the knowledge I then had of this disease, I must freely confess that I should have been tempted to advise amputation had the disease been limited to one side. As it was, I had but little hope of saving the patient's life. Contrary to all expectation, however, slow but uninterrupted improvement began to take place. A slight effusion reappeared in a few days but was absorbed without further treatment after several weeks. A number of months subsequently, several rough pieces of bone escaped through the openings, after which suppuration ceased and the wounds closed. About a year after the beginning of the disease the patient was able to walk with the aid of a cane, the joints being somewhat stiff. At present the upper extremities of both tibiæ are enlarged, the mobility of the joints is completely restored, the patient is a strong, healthy young man, nothing in his gait indicating the extent of his previous disease.

CASE XII. *Osteo-myelitis of tibia; partial separation of lower epiphysis; operation; recovery.*—August Scholtke, aged 11 years, a previously healthy boy, was taken with a severe pain in his right leg, accompanied by grave constitutional symptoms during the month of January, 1879. In a number of weeks an abscess opened spontaneously above the ankle-joint after which the pain diminished but the patient remained in bed in a feeble condition for several months. He was admitted to the Milwaukee Hospital, August 15, 1879. On admission the lower part of the tibia was very much enlarged from the ankle-joint to the juncture of the middle with the upper third. Three fistulous openings existed, as follows: over the middle of the shaft, above the internal malleolus, and above and internal to the external malleolus. With a probe a loose piece of bone was discovered, surrounded by a strong involucrum. After placing the patient under the influence of ether and applying Esmarch's bandage, an operation under antiseptic precautions was performed for the removal of the sequestrum. With a chisel the cloacæ were sufficiently enlarged to permit the extraction of the sequestrum, which measured 18 ctm. in length and was rough on all sides, indicating its central origin. A partial separation of the lower epiphysis was found; a probe could be passed freely in every direction between it and what remained of the shaft.

The granulations were scraped out with a sharp spoon and the cavity disinfected with an 8 per cent. solution of chloride of zinc and carbolized water. Lister's dressing was applied. No fever followed the operation and only a trace of suppuration. The process of repair was initiated at once and in a few weeks the wound had entirely healed. The new tibia is 1 ctm. longer than the opposite one, giving to the foot a slight varus appearance.

CASE XIII. *Osteo-myelitis of the tibia; separation of upper epiphysis; recovery.*—John M., aged 12 years, during the month of March, 1871, while engaged playing ball, was suddenly siezed with an intense pain in the head of the right tibia, which soon became so severe that he was unable to stand on the affected limb. Slight fever was present from the beginning, and a diffuse swelling appeared below the knee in a few days. In about three weeks an abscess was opened below the knee which gave exit to a considerable quantity of osteo-myelitic pus. Antiseptic injections were made daily and the wound covered with a gauze compress kept saturated with carbolized water. About two weeks after the incision was made, complete epiphyseolysis and swelling of the knee-joint took place, but as the fever had almost entirely subsided, the effusion was supposed to be of a serous nature. A plaster of Paris bandage was applied from the toes to the groin with a fenestrum over the incision. Profuse suppuration for a while threatened to prostrate the patient but diminished after the removal of a rough, thick but short sequestrum. The effusion into the joint disappeared without special treatment and the patient regained his usual health and almost perfect use of the limb in a little over a year. Soon after the separation of the epiphysis the the patient was seen by an old surgeon, who advised amputation as the only means of saving his life; having, however, observed the wonderful restorative power of the *vis medicatrix naturæ* in similar cases, I insisted on pursuing a conservative course and was amply rewarded by obtaining such a satisfactory result.

From anatomico-pathological reasons the result is less fortunate if separation of the upper epiphysis of the femur or humerus takes place during an attack of osteo-myelitis. In these cases the head of the bone, deprived of its principal blood supply and surrounded by the products of inflammation, becomes necrosed and acts as a foreign body, exciting, not unfrequently, serious disorganization of the remaining joint structures, as was the case in the following instance:

CASE XIV. *Osteo-myelitis of femur; separation of upper epiphysis; operation; recovery.*—L. C., aged 10 years; the youngest member of a healthy family of children, fell from a haystack during the summer of 1870, and, as was thought at the time, sprained his hip. He was soon able to leave his bed, but walked quite lame; a few weeks later the knee on the same side began to pain him; the hip and upper part of the thigh became swollen and tender; violent constitutional symptoms followed, with hectic fever and rapid emaciation. In about three months an abscess formed in front of the upper part of the thigh, which soon opened spontaneously and discharged a large quantity of pus. The patient remained in a very feeble condition for several months, when gradual improvement commenced to take place, so that in the spring he was able to walk on crutches. About eighteen months later I performed excision of the hip-joint. The head of the bone was found loose in the joint; on making a section through the bone below the great trachanter, the bone was found very soft and the cancellæ infiltrated with pus, the sawed ends presenting a greenish or gray color; consequently two more sections were made lower down until healthy bone was reached, removing in all the head and four inches of the shaft. The periosteum was thickened, and was carefully separated and preserved. The wound closed in a short time, but reopened on several occasions during the next two years, discharging several small spiculæ of bone, when it finally closed permanently, and the patient recovered with a very useful limb—motion in the false joint and two inches of shortening.

CASE XV. *Osteo-myelits of femur; separation of upper epiphysis; operation; recovery.*—George Muehlhauser, aged 15 years, of a strumous diathesis, has been in delicate health for a number of years. Without any obvious cause, he began to suffer from symptoms of acute coxitis in the fall of 1875. For several weeks he was treated on general principles, rest, extension, fixation, tr. iod., fomentations, etc., when the general symptoms became so grave that excision of the head of femur was considered the only chance to save the patient's life. On opening the joint, the head of the femur was found necrosed and completely detached, the upper portion of the femur was soft and infiltrated with pus. On a level with the trochanter minor the bone was porous, otherwise healthy. In about a year the patient recovered completely, the limb being about 4 ctm. shorter. In both of these cases the disease originated evidently in the cancellous tissue near the cartilage of agglutination and involved the joint secondarily. In the last case the upper and posterior portion of the acetabulum was denuded of cartilage, the affected tissues were scraped out with a sharp spoon. The prognosis in regard to the usefulness of a limb after an attack of osteo-myelitis depends less upon the extent of bone involved than the presence and severity of joint complications.

*Pathology.*—Simple non-infectious inflammation of the medulla is a physiological reparative process. According to Virchow, the medulla of infants resembles granulation tissue chemically and microscopically. The restorative function of myeloid tissue is studied most advantageously in cases of fracture and around osteomyelitic foci; in these instances its cells are transformed directly into bone cells, uniting the fragments in the former case and limiting the extension of the latter. In osteo-myelitis the inflammatory process extends beyond the process of repair, its tendency being in an opposite direction—to suppuration and necrosis. The inflammation is a specific one, and depends upon the presence of septic germs which are either brought in direct contact with the medulla or have gained entrance from without into the circulation through the respiratory or gastro-intestinal tract, and are conveyed with the blood to the marrow where localization takes place. Many causes which have been previously enumerated may determine localization, but the *sine quâ non* factor in calling into existence this destructive process, is the presence of living septic bacteria.

*Morbid Anatomy.*—Lidell describes three stages or morbid conditions in which the marrow may be found: 1. Carnification, 2. Suppuration, 3. Gangrene. In the first stage the substance of the marrow appears congested and has become abnormally firm. Fat globules are set free and are removed and the myeloid cells are generated with great rapidity. The sclerosis of the medulla is owing to a production of new connective tissue and partly to a hardening of the existing fibrous network. Only in exceptional cases is the disease arrested in this stage, but usually passes into the stage of suppuration. Acute œdema of the cellular tissue around the bone indicates that suppuration has taken place in its interior. Generally multiple deposits are disseminated over a greater or less area of marrow, but two or more of these purulent collections may coalesce and form large suppurating cavities. Pus globules are produced from leucocytes, connective tissue or myeloid cells, or all of them. In the cancellated tissue the cancellæ are either removed by pressure and absorption, forming abscess cavities, or a certain district loses its vitality and is separated from the surrounding living tissue as a sequestrum. The most intense form of infection may terminate in gangrene of the marrow. This form is extremely rare, and when it does occur, it is in traumatic cases, after amputations, in infected and over crowded hospitals. As a direct extension of the morbid process to adjacent tissues by continuity or contiguity of structure, we have present in every case, ostitis, osteo-chondritis, periostitis and in most cases participation of neighboring joints.

The bone substance, from the intensity of the inflammatory process, or from arrest of circulation, undergoes necrosis, and if the life of the patient is preserved, is sooner or later detached from the living tissues. Instances have been reported where an entire bone, including both articular ends, has lost its vitality, constituting a large sequestrum. Usually only a part of the shaft, and in preference that near one of the ends of the diaphysis, becomes necrosed. In the lower part of the femur, necrosis takes place most frequently on the posterior aspect of the bone, and in the upper extremity of the humerus on the outer surface. If the vitality of the bone is not destroyed, its substance is softened by liquefaction and absorption of the inorganic matter, and from increased histogenesis of the marrow and bone cells which subsequently produce sclerosis. Osteo-chondritis may exist as a distinct affection, but more frequently it constitutes an important element in the history of osteo-myelitis. It may result in a partial or complete separation of the epiphysis from the diaphysis. I have no doubt that the disk of temporary cartilage placed between the shaft and epiphysis, serves a useful purpose in protecting the joint from the invasion of disease. The cartilage interrupts completely the continuity of the medullary tissue in the epiphysis and diaphysis, and to a less extent also the vascular connections. If the disease originates in the diaphysis, the cartilage must be destroyed to a greater or less extent before it can invade the epiphysis. Mr. Hutchinson, in his "Illustrations of Clinical Surgery," gives a beautiful illustration to support this view. The femur was amputated at its upper third. The patient died of pyæmia. The marrow and cancellated tissue of the bone were infiltrated with pus and presented a greenish color from the end of the bone to the cartilage of the great trochanter and head of bone. The bone above the cartilages was perfectly normal. I believe it is also a well-established fact that adults are more liable than children to suffer from destructive suppurative synovitis of joints, if the disease is located near the articular ends of the bones.

The softening of the cartilage usually commences at its margins. After complete or partial destruction of its substance, if the disease has not expended its force, the epiphysis is in danger of becoming infiltrated with the infectious products of inflammation, and the joint is placed in great jeopardy. Joint affections usually make their appearance from the 15th-19th day (Chassaignac), but may occur as early as the third day, or be delayed for months in chronic cases. The swelling of the joint appears slowly and insidiously, without any additional severe local or general symptoms. The joint becomes affected either by the establishment of a direct communication with the osteo-myelitic center through a perforation of the articular cartilage, or by an extension of the periosteal inflammation to the structures of the joint.

In some instances a chronic osteo-myelitis in an epiphysis leads to caseous degeneration of the products of inflammation (phthisis of bone) which may remain latent for a long time, but when it begins to break down and suppuration takes place, perforation into the joint is a frequent occurrence. If the joint communicates directly with the disease in epiphysis, the result is necessarily a suppurative synovitis with extensive disorganization of the articular and peri-articular tissues, and may produce infection of the opposite bone. Mr. Hutchinson, in his work quoted above, reports a very interesting case of this kind. A boy who had suffered a contusion of the hip, died of pyæmia two weeks after the injury. A post mortem examination revealed the existence of dirty pus in the hip-joint. The iliac bone and head of femur showed signs of recent inflammation. The affected portions of bone were discolored, being yellow in some parts, dusky purple or green in others. The discoloration in the head of femur was abruptly limited by the line of the epiphyseal cartilage.

If the joint inflames by contiguity, the process extending from the periosteum to the ligaments and synovial membrane, the product of inflammation is more likely to be serous, or sero-purulent, and the function of the joint is less apt to suffer permanent injury. Entrance of pus into a joint between the periosteum and bone must be a rare accident, as this membrane, with the ligaments is very firmly attached to the surface of the epiphysis. The periosteum participates, to a greater or less degree, in all cases. The periostitis must be purulent, fibrous, hæmorrhagic or osteoplastic, according to the character of the exudations. The disease extends to the periosteum from the primary seat through the Haversian canals or at the epiphysary junction. The irritation may not exceed the physiological limits and production of bone is the result. In severe and acute cases suppuration is always the result. The hæmorrhagic form is invariably fatal. If an incision is made before perforation of this membrane has taken place, its outer surface is recognized by its very dark brown, purple or almost black color. In typical cases the pus underneath it has a brown, bluish, emulsion-like aspect. After perforation of the periosteum, pus escapes into the parostral tissues and produces the well-known phlegmonous inflammation of the connective tissue. If the entire marrow has been destroyed and the periosteum completely separated, circulation in the bone is arrested and necrosis of the entire diaphysis is the inevitable result. After the acute symptoms have subsided, the reparative process is at once inaugurated. New bone is formed on the inner surface of the periosteum, which forms a case or involucrum around the dead bone until the latter is detached and removed as a sequestrum, either by spontaneous expulsion or through the interference of art. The cloacæ which lead to the dead bone indicate deficiencies in the periosteum, caused by spontaneous perforation, incision or destruction of the membrane. Osteo-sclerosis follows osteo-myelitis, but in exceptional cases the new bone remains porous and soft (osteo-porotic), a condition described by Volkmann and Schede, which may become the cause of various degrees of deformity from bending of the shaft. The veins of the marrow, bone and periosteum in inflammation of these structures are most always affected; the exceptions to this rule are few. Coagulation of the blood in the veins leads to phlebo-thrombosis. If the clot fails to undergo organization, it breaks down and small particles enter the circulation and are carried to distant organs where they are arrested as embola, causing metastatic deposits so characteristic of pyæmia. The same result may follow phlebitis and periphlebitis.

Dr. Woodward attributes the frequency with which pyæmia occurs in this disease to a septic infection which is propagated by continuity from the putrefied marrow to coagula in the veins.

Hutchinson believes that the particles of organic matter not only produce embolism and thrombosis but that they serve at the same time as carriers of living matter capable of infecting the tissues around the plugs.

*Treatment.*—The indications to be fulfilled in the treatment of a case of osteo-myelitis are:

1. To neutralize, if possible, the infectious element by local and general use of antiseptics. 2. To subdue inflammatory action. 3. To establish, under antiseptic precautions, an early and efficient outlet for the products of inflammation. 4. To prevent deformity. 5. To sustain the patient's strength by procuring the best hygienic surroundings and by the early administration of stimulants and tonics.

An early diagnosis is of paramount importance. An uncertainty in this regard often prevents the adoption of timely and positive measures to prevent or combat the progress of the disease. The deep-seated location of the malady offers the greatest obstacle to a correct diagnosis and successful treatment. Taking it for granted that in the spontaneous variety the septic germs enter the system through the gastro-intestinal tract it would be rational to assume that a brisk cathartic administered soon after the appearance of the first symptoms would be an efficient remedy to cut off any further supply of these noxious agents. In many instances nature makes an attempt in this direction by establishing a copious diarrhœa. A large dose of calomel administered for the same purpose and in the same manner as advised during the early stage of typhoid fever, could not fail to produce a beneficial effect. Salicylate of soda given in large doses from 6.0—24.0 grms. during 24 hours has been advised by Kocher with a view to neutralize the poison. In such doses this remedy would also promptly reduce the temperature, which is constantly high in all acute cases. If the medicine is rejected by the stomach it can be administered by enema. Opium must be given in sufficient doses to alleviate pain. The limb should be placed in an elevated position and the strong tr. iodine applied daily until vesication is produced. Demme, Billroth and Volkmann speak very highly in favor of iodine applications. If there is reason to believe that the first stage has not passed, ice should be applied over the part affected for the purpose of diminishing congestion, provided such applications are agreeable to the patients. As soon as swelling takes place it is a sign that suppuration has taken place when warm fomentations should be substituted.

In regard to incisions, great diversity of opinion still prevails. Previous to the researches of Demme, early and free incisions were practiced. As the results in most cases were disastrous, he was led to adopt a more conservative treatment. He resorted to an expectant course of treatment until the poison had exhausted itself in the system, as indicated by reduction of temperature and cessation of local inflammation, and then made large incisions. For the purpose of affording an outlet to the products of inflammation, Klose made early and small incisions over the epiphysary junction.

Most all authorities agree that early and free incisions expose the patient to additional risks of septicæmia and pyæmia, while at the same time the primary seat of suppuration is not reached unless trephining is also performed.

M. Ollier is an advocate of early incision combined with trephining. In a communication read before the Academy of Sciences of Paris, he claims that trephining is applicable to all forms of osteo-myelitis with severe general symptoms. He says the most painful variety is usually found near the extremities of long bones. Trephining, even in the most diffuse form, will arrest the intense pain by reliving pressure ; where the disease is circumscribed it affords prompt and decided relief. In the acute form, trephining will often prevent the grave and fatal symptoms, while in the sub-acute and chronic form it removes the most distressing symptom—pain. In eight out of nineteen cases of painful osteo-myelitis he found pus ; and in ten cases the marrow presented different diverse morbid appearances, while in the last case, a case of acute osteo-myelitis of femur, a large quantity of fluid blood escaped. Two of the nineteen cases died of pyæmia. After pus has appeared beneath the periosteum or in the parosteal tissues, no time should be lost to remove it promptly. If it is permitted to accumulate, it may seriously compromise the integrity of neighboring tissues and organs and in addition serve as a source of infection. It can be removed safely with the aspirator and the abscess cavity thoroughly washed out with carbolized water for the purpose of neutralizing the local infection, or by making one or more incisions under antiseptic cautels. Lister has found that no less than a five per cent. solution of carbolic acid is necessary to destroy bacteria or their products. Hueter has employed parenchymatous injections of carbolic acid with decided benefit in other inflammatory affections of bones and soft tissues. Kocher suggests injections of carbolized water into the tissues around the bone, made with a hypodermic syringe, several syringes full to be injected at different points twice daily. It seems to me the safest plan would be, after fluctuation can be detected, to remove the fluid with an aspirator, using a large needle and subsequently washing out the cavity with a five per cent. solution of carbolic acid. This simple procedure should be repeated as soon as the fluid reappears and until the acute local and general symptoms have subsided, when a permanent opening can be made without much risk of secondary infection. Kocher also suggests the propriety of making intra-osseous injections by penetrating the bone with a small perferator, and injecting carbolized water, thus reaching the primary focus of the disease. Theoretically the suggestion appears valuable, practically it would be uncertain and difficult of execution. Incisions, especially if practiced during the acute stage, should be made only under the antiseptic treatment. They should be made, if possible, at a point out of reach of any important vessels and nerves and over some well marked intermuscular septum. The limb should be shaved, cleaned and disinfected around its entire circumference and over a considerable space. To prevent hæmorrhage the knife should be used as little as possible; after dividing the skin and fascia the handle of scalpel and finger should be used to separate the muscles until the pus escapes, or if the periosteum remains intact it is incised with the knife. The opening should be sufficiently large to permit the introduction of the index finger for the purpose of ascertaining the condition of the bone. If necessary one or more counter openings must be made, with a view of securing free drainage. Large drainage tubes should be inserted, the cavity thoroughly washed out with a weak solution of chloride of zinc or a five per cent. solution of carbolic acid and the antiseptic dressing applied.

No special benefit can accrue from making the incisions 12–30 ctm. in length as recommended and practiced by Demme. The antiseptic treatment of wounds justifies operative interference, which would heretofore have been considered unsafe and imprudent. It prevents decomposition of the products of inflammation and its result—septicæmia. Should putrefaction take place, then an attempt should be made to render the abscess cavity aseptic by applying thoroughly an eight per cent. solution of chloride of zinc, followed by a five per cent. solution of carbolic acid. Should an opening exist before the patient comes under treatment, free drainage must be established by enlarging the opening or making new incisions. Where the greater portion or the entire diaphysis of a bone has become necrosed and has become separated at one or both epiphysary junctions, it may become necessary to remove it during the acute stage to avert exhaustion of the patient from the profuse discharges and hectic irritation incident to such a condition. It has been argued against this operation, that the bone would not be sufficiently regenerated after its removal. This fear is however not supported by facts. Duplay, Holmes, McDougal, Lefort, Giraldes, Spence, Petregrin, Philzky, Wilms, Cheever, Ropes, and Gay, have each reported cases where almost complete reproduction followed the removal of the entire shaft. It is very important, especially in young persons, to preserve both epiphyses to prevent subsequent shortening and deformity of the limb.

I will report one case, where, after excision of two thirds of the shaft of the tibia, complete reproduction took place.

CASE XVI. *Osteo-myelitis of tibia ; excision of two-thirds of the shaft; recovery.*—John Endlich, aged 12 years, of healthy parents, was the subject of osteo-myelitis of the right tibia which resulted, after four weeks, in separation of the upper epipysary junction; the knee-joint was considerably swollen at the time. The temperature was continuously high, delirium, rapid emaciation, etc. An incision was made three inches below the knee, after the second week, which gave exit to a large quantity of thin sanious pus on which floated numerous fat globules. Six weeks from the commencement of the attack the constant fever and profuse suppuration threatened fatal exhaustion unless the source of irritation could be removed; it was consequently decided to perform excision of the shaft. After the patient was placed under the influence of chloroform an incision was made along the inner surface of the tibia, extending along the entire length of the diaphysis. The upper epiphysis was found detached, and the upper two-thirds of the shaft denuded of its periosteum; the bone was divided with a chain saw, about two inches above the ankle-joint. Immediately above the section the lines of demarcation between the dead and living bone could be distinctly seen. The medullary canal of the portion of bone removed was infiltrated with pus. The inner surface of the periosteum showed, at some points, deposits of new bone. The limb was placed at first upon a posterior splint, and afterward a fenestrated plaster of Paris splint was applied from the toes to the knee, and the wound dressed with carbolized oil. The fever and discharge disappeared almost entirely after the operation, and the general health began to improve. New bone began to fill up the large gap and in four months complete regeneration had taken place. The tibia is now somewhat larger and longer than the opposite one and the function of the leg is perfect. When both epiphyses have become separated from the diaphysis, the operation for removing the shaft is an easy one; all that is necessary is to make an incision sufficiently large to remove the sequestrum. Suitable mechanical support must be applied for a long time to prevent bending of the new bone. Should swelling of one or more joints arise, it is always well to make an exploratory puncture with a hypodermic syringe to ascertain the character of the effusion. If the effusion is serous no special treatment is necessary, unless the tension becomes great causing severe pain, when it can be removed by simple aspiration. If it is found sero-purulent, it should be removed with aspirator and the joint washed out with a 5 per cent. solution of carbolic acid, and the operation repeated as often as may become necessary. If the joint contains pus the same treatment is applicable, but if the effusion reappears in a short time then incisions and drainage, according to Lister's directions, offer the best prospects for recovery. This latter treatment is positively indicated if the joint communicates directly with the disease in the bone, as in such a case perforation of the articular cartilage and serious destruction of other joint tissues has taken place. During the septic stage of acute osteo-myelitis with suppurative synovitis, amputation may become necessary to save the life of the patient. In exceptional cases the same sad alternative may be required after the acute symptoms have subsided for the purpose of removing the source of exhausting suppurative discharges. Our present means of treating abscesses and diseases of joints are fortunately sufficiently advanced so that even severe cases can be safely treated on a conservative plan, and amputation must be reserved only as a *dernier ressort* in extreme cases.

In chronic cases the articular extremities are usually the seat of disease, and as there is great danger that the joint may become involved directly by perforation of the cartilage, they should receive early attention and thorough treatment. If the bone is enlarged, softened, and no external opening exists, excellent results are obtained by ignipuncture, an operation introduced by Richet. The part affected, and considerable of the surface beyond, should be prepared in a similar manner as for incision; while the field for operation is covered with a spray, the round, sharp platina point of Paquelin's thermo-cautery, heated to a white heat, should be thrust into the interior of the bone at several points, and Lister's dressing applied. If an opening exists, an incision should be made, the cloaca enlarged, and the diseased and infected tissues in the interior of the bone thoroughly removed with a sharp spoon.

If the joint has become affected by suppurative synovitis, the choice can only be between resection and amputation. The special indications of each individual case must determine in favor of one or the other of these operations.

From the frequency with which the joints become affected during the progress of this disease, our attention should be directed early towards proper management of this complication. Deformity should be prevented by appropriate mechanical support by means of splints or plaster of Paris dressings. A retentive apparatus, combined with suspension, will not only prevent contractures, partial dislocations of joints, and bending of newlyformed bone, but will add materially to the comfort of the patient.

Should signs of pyæmia arise, our main reliance must be placed on the administration of large doses of quinine and alcohol. Luecke has obtained the best results from the administration of large doses of alcohol. Instances have been reported where two bottles of cognac were given, with decided benefit, during twenty-four hours. The patient should be surrounded by the most favorable hygienic influences, as fresh air, equable temperature, light and an abundance of plain, nutritious food. As soon as the acute symptoms have subsided, iron, especially the tr. ferri chloridi, should be freely administered. Should any dyscrasia, as syphilis, scrofula, tuberculosis or scorbutus, exist, it should be corrected by appropriate medication.

### CONCLUSIONS.

1. Spontaneous osteo-myelitis is an infectious disease.

2. It is most prevalent in damp, changeable climates, and during the winter and spring months.

3. It affects with preference individuals during the period of growth and development of bone.

4. Traumatism and other agencies, which produce a retardation or arrest of circulation in the vessels of the marrow, act only as determining causes.

5. Its primary seat is usually in the marrow of the cancellated tissue, in close proximity to the epiphysary cartilage.

6. Joint affections are frequent and prominent complications of this disease.

7. Thrombosis and inflammation of the veins of the marrow, bone, periosteum and soft parts are of frequent occurrence, and are the direct cause of pyæmia.

8. Swelling is absent for the first few days, and when it does occur, it becomes rapidly diffuse, and is attended by œdema and enlargement of the superficial veins.

9. Fluctuation is diffuse as soon as its existence can be ascertained.

10. A constant high temperature and typhoid symptoms indicate the gravest type of the disease.

11. Death may result from the intensity of the primary infection, but is usually produced by some complication.

12. Early removal of the products of inflammation, under strictest antiseptic precautions and local disinfection of the tissues, are of paramount importance for its successful treatment.

13. Epiphyseolysis may become completely repaired.

14. Excision of shaft may become necessary during the acute stage, to prevent exhaustion from profuse suppuration ; this rule is not applicable if the humerus or femur is affected, on account of the impossibility of keeping the limb in position until regeneration of bone has taken place.

15. In most cases fixation of the limb is necessary for the purpose of procuring rest and to prevent deformity.

AUTHORITIES REFERRED TO.

Allen, *American Jour. Med. Sciences*, July, 1865.

Auger, *The Monthly Abstract of Med. Science*, June, 1879.

Azam, *Schmidt's Jahrbuecher*, Band 123, S. 204.

Bardeleben, Lehrbuch der Chirurgie u. Operationslehre. Berlin, 1871.

Billroth, Surgical Pathology. New York, 1872.

Demme, *Schmidt's Jahrbuecher*, Band 119, S. 204.

Demme, *Schmidt's Jahrbuecher*, Band 124, S. 55.

Emmert, Lehrbuch der Chirurgie. Stuttgart, 1850.

English, *Schmidt's Jahrbuecher*, Band 149, S. 187.

Erichsen, Science and Art of Surgery. Philadelphia, 1869.

Frey, Histologie u. Histochemie des Menschen. Leipzig, 1874.

Gay, Excision of the Diaphysis of Tibia. Transactions American Med. Association, 1878.

Gosselin, Clinical Lectures on Surgery, 1878.

Gosselin, *The Monthly Abstract of Med. Science*, August, 1879.

Goltdammer, *Berliner Klinische Wochenschrift*, May, 1877.

Green, An Introduction to Pathology and Morbid Anatomy.

Guersant, Clinical Lectures on Surgery.

Heinecke, Necrosis, *Volkmann's Klinische Vortraege*, Band 1.

Helferich, Ueber die nach Nekrose an der Diaphyse der langen Extremitätenknochen auftretenden Störungen im Laengen wachsthum derselben. *Deutsche Zeitschrift fuer Chirurgie*, Band X.

Holmes, A System of Surgery.

Hueter, Klinik der Gelenkkrankheiten.

Hutchinson, Illustrations of Clinical Surgery, 1879.

Koenig, Lehrbuch der Chirurgie. Berlin, 1879.

Kocher, Die Acute Osteo-myelitis mit besonderer Ruecksicht auf ihre Ursachen. *Deutsche Zeitschrift fuer Chirurgie*. Band XI, Heft. 1 u. 2, 3 u. 4.

Kocher, *Volkmann's Klinische Vortraege*, No. 102.

Lidell, United States Sanitary Commission Memoirs, Vol. I.

Ollier, *The Monthly Abstract of Med. Sciences*, March, 1877.

Paget, Surgical Pathology.

Ritter, *Schmidt's Jahrbuecher*, Band 172, S. 160.

Rosenbach, Beitraege zur Kenntniss der Osteo-myelitis. *Deutsche Zeitschrift fur Chirurgie*, Band X, Heft. 3 u. 4, 5 u. 6.

Roser, Handbuch der Anatomischen Chirurgie, Tuebingen, 1868.

Senn, Necrosis and its Treatment. Transactions Wisconsin State Med. Society, 1872.

Smith, The Pathology of the Bones. Transactions American Med. Association, 1878.

Stanley, A Treatise on Diseases of the Bones. Philadelphia, 1849.

Virchow, Cellular Pathology.

Volkmann, Ueber den Charakter u. die Behandlung der fungoesen Gelenkentzuendung. *Klinische Vortraege*, No. 168 u. 169.

Volkmann, Osteo-myelitis, VonPitha u. Billroth's Handbuch der Allgemeinen u. Speciellen Chirurgie.

Wahl, *Schmidt's Jahrbuecher*, Band 133, S. 324.

Weinbechner, Resection of entire Diaphysis of Tibia. *American Journal of Medical Sciences*, Oct., 1879.

Woodward, Pyæmia. Transactions American Med. Ass., 1869.

